# 

**Published:** 1860-10

**Authors:** 


					﻿Published Monthly, at $3 per Annum in Advance.
w
0$
h
V
z
s
o
g
x
a
■
u
&
w
©
-
M
X
©
w
-
S
' ©
«
a
•
u
a
>
■
■-
x
> ©
>•
5
i
w
©
©
M
aT
©
g
©
©
»o
I
0
•*
h
s
e
X
i
•*
©
us
X
©
S
©
©
1 *®
©
©
•O
s
d
:
B
«
9
LL1 T j -Z\_ ZTxT -ZX_
11® M swat
JOURNAL.
/. G. WESTMORELAND, M. D.,
Professor <if Materia Madica axd Medical Jurisprudence in tue Atlanta Medical Colleor,
EDITOR AND PROPRIETOR.
Phx et .cientia, ued verltaw wine timore.
Vol. VI.	OCTOBER, 1860.	No. II.
ATLANTA, GA:
ATLANTA IJSTTEIaIaIGK^CER PRINT.
I 860.
Each Number contain. Sixty-Four Page.
•a I
9
X
e*
B 1
?
•*
3
®
i
s •
©
x
® I
& .
0
0
©
B
©
J
;l
9
X
c
M
K
S I
R
S
x
5
r*
t»
9
t
9
9
S
r»
B*
®
0
a
•<
■B
. a
—
x
5
?
a
»
M
t»
9
9
%
9
3
o
o
a
				

